# Diagnostic accuracy of real-time polymerase chain reaction assay for the detection of *Trichomonas vaginalis* in clinical samples: A systematic review and meta-analysis

**DOI:** 10.4102/ajlm.v14i1.2522

**Published:** 2025-04-16

**Authors:** Emmanuel O. Babafemi, Benny P. Cherian, Khalid Rahman, Gilbert M. Mogoko, Oluwatoyin O. Abiola

**Affiliations:** 1 Department of Pharmacy and Biomolecular Sciences, Faculty of Science, Liverpool John Moores University, Liverpool, United Kingdom; 2 Department of Microbiology, Royal London Hospital, Barts Health NHS Trust, London, United Kingdom; 3 Department of Microbiology, IPP Pathology First, Dobson House, Bentalls, Basildon, United Kingdom; 4 Department of Computer Science, Faculty of Science, Afe Babalola University, Ado Ekiti, Nigeria

**Keywords:** Trichomoniasis, *Trichomonas vaginalis*, real-time polymerase chain reaction assay, vaginal swabs, systematic review, meta-analysis

## Abstract

**Background:**

Vaginal trichomoniasis is a highly prevalent parasitic infection associated with HIV acquisition and preterm birth. The ‘gold standard’ for its diagnosis requires 3–7 days to detect by culture. Rapid and accurate diagnosis, such as by nucleic acid amplification testing, is key to manage the disease, and control and prevent its transmission.

**Aim:**

This review aimed to assess the overall accuracy of real-time polymerase chain reaction (RT-PCR)-based assays, for routine diagnosis of *Trichomonas vaginalis* in clinical vaginal samples from women with symptomatic/asymptomatic trichomoniasis, using Trichomonads culture as the gold standard.

**Methods:**

MEDLINE, PubMed, EMBASE, and other sources were used to search for included studies published between 01 January 1995 and 31 July 2023. The search terms ‘real-time polymerase chain reaction’, ‘real-time’, ‘polymerase chain reaction’, ‘*Trichomonas vaginalis*’, ‘trichomonas’, ‘vaginalis’, ‘humans’, ‘rt pcr’, ‘nucleic acid amplification test’, ‘NAAT’, ‘trichomonad culture’, ‘women’ were included. Summary estimates were calculated for the overall accuracy of the assay compared to Trichomonads culture as the reference standard. Meta-analysis was conducted using a bivariate meta-regression model.

**Results:**

Twenty-seven eligible studies met our inclusion criteria: sensitivity 99% (95% confidence interval [CI] 99–100), specificity 100% (95% CI 100–100), positive likelihood ratio 350.67 (167.42–734.49), negative likelihood ratio 0.02 (0.01–0.03), diagnostic odds ratio 23 064.05 (95% CI 8532.13–62 346.77), and area under receiver operating characteristics curve 0.99. There was significant heterogeneity in sensitivity and specificity (*p* < 0.001).

**Conclusion:**

Our results suggested that RT-PCR assays could be useful for the diagnosis of vaginal trichomoniasis with high sensitivity and specificity.

**What this study adds:**

This article provides a comprehensive review of the effectiveness of RT-PCR assays for the diagnosis of trichomoniasis with high sensitivity and specificity in comparison to other methods in clinical laboratory practice. The goal is to present awareness/evidence that this assay is more accurate and rapid than other techniques.

## Introduction

*Trichomonas vaginalis*, a protozoan, causes trichomoniasis, which is a common sexually transmitted disease (STD) that affects approximately 156 million persons globally per year, with the majority in low-income settings.^1^ It has been estimated that 7.4 million new cases occur annually in the United States.^2^ *Trichomonas* is the most common non-viral STD agent in the world, with an overall prevalence of 3.1%.^3^ Trichomoniasis occurs in both men and women, causing infection; however, symptoms are widespread in women. Symptomatic women present a malodorous, diffuse, vulvar irritation, with yellow-green vaginal discharge that may be mistaken for bacterial vaginosis. *Trichomonas vaginalis* is known to cause vaginitis, cervicitis, and many infections that may go symptomless, with likely consequences such as premature birth, underweight at birth, tubal infertility, and pelvic inflammatory disease when left untreated.^4^ Trichomoniasis may lead to adverse birth outcomes, such as increased risk and transmission of HIV infection, and premature rupture of the membranes, in women.^5^ Hormonal changes predispose to a higher incidence of lower genital tract infections caused by trichomoniasis during pregnancy, which can lead to perinatal and maternal complications.^6^

Tests with improved sensitivity and specificity are of great significance and essential for diagnosing trichomoniasis. The gold standard for the diagnosis of *T. vaginalis* infection is culture of the organism using vaginal specimens and is reported to have 75% to 89% sensitivity; however, it requires between 2 days and 7 days of incubation, resulting in substantial delays before obtaining the results.^7^ Direct microscopic examination of the vaginal fluid using wet preparations remains the most widely utilised diagnostic test for *T. vaginalis* infection, despite its limited sensitivity in asymptomatic patients.^8^ Microscopic examination is inexpensive and quick, but depends on the microscopist’s skill, and on the prompt transport and processing of the sample, which relies on the viable organisms, to avoid the loss of parasite motility.^9^

In addition, several authors have reported the use of nucleic acid amplification tests, including real-time polymerase chain reaction (RT-PCR). These have shown an improved sensitivity and specificity method for detecting *T. vaginalis* compared to microscopic examinations and culture.^10,11^ Real-time PCR assays provide an improvement in medical screening for the parasite.^11^ Therefore, a simple, rapid, and accurate diagnostic test with acceptable sensitivity and specificity is important in diagnosing *T. vaginalis* infection. This cannot be accurately diagnosed based on the clinical picture, because clinical symptoms of trichomoniasis may be similar to those of other STDs.^12^

All the available published primary research studies were used in this review to provide summary estimates of the diagnostic accuracy of RT-PCR assay for detecting *T. vaginalis* from clinical samples. The study summarises current evidence-based clinical practice that can help diagnose *T. vaginalis* during pregnancy to prevent perinatal and maternal complications. The findings will help in choosing the most appropriate tool for rapid and accurate detection of *T. vaginalis* in pathological samples on a routine basis, medical screening, future guidelines and healthcare policy.

## Methods

### Study protocols

The Preferred Reporting Items for Systematic Reviews and Meta-Analyses statement guidelines^13^ were followed to conduct this systematic review and meta-analysis (Online Supplementary Text 1). The Preferred Reporting Items for Systematic Reviews and Meta-Analyses checklist was used to ensure that all the relevant information from studies (published 01 January 1995 to 31 July 2023, in any language) and unpublished articles was eligible to identify trichomoniasis among women in the analysis. We registered our systematic review protocol in PROSPERO (International Prospective Register of Systematic Reviews): PROSPERO CRD42023435253.

The Quality Assessment of Diagnostic Accuracy Studies-2 (QUADAS-2)^14^ was used to assess the quality of the included studies. There was no need for institutional ethical review approval for this study.

### Searching strategies

The search was conducted with the aid of carefully selected terms. The search strategy included ‘real-time polymerase chain reaction’, ‘real-time’, ‘polymerase chain reaction’, ‘*Trichomonas vaginalis*’, ‘trichomonas’, ‘vaginalis’, ‘humans’, ‘rt pcr’, ‘nuclei acid amplification test’, ‘NAAT’, ‘trichomonad culture’, and ‘women’. They were used distinctly and in combination, using Boolean operators such as ‘OR’ or ‘AND’ to generate a list of primary studies. There was no language limitation to the search. A librarian information specialist familiar with the topic validated the search strategy for each database. Two of the investigators independently and systematically searched the electronic bibliographic databases (MEDLINE, PubMed, EMBASE, and other relevant databases) to identify additional records^15,16^ (Online Supplementary Text 2). ‘Google Translate’ was used to screen abstracts and articles in languages other than English.

### Eligibility criteria

Observational studies (cross-sectional and cohort) and case-control designs for detecting *T. vaginalis* from women clinical samples of any age were included.

The studies were eligible for inclusion if they reported the total number of patients tested, described original research, contained positive/negative results that allowed the calculation of true positives, true negatives, false positives, and false negatives, and compared RT-PCR assay to a reference/gold standard method – culture-based assay.

Exclusion criteria included studies where RT-PCR assay was not used, *T. vaginalis* was detected in men, involvement of animals, and duplicate publication.

### Study selection process

**FIGURE 1:**
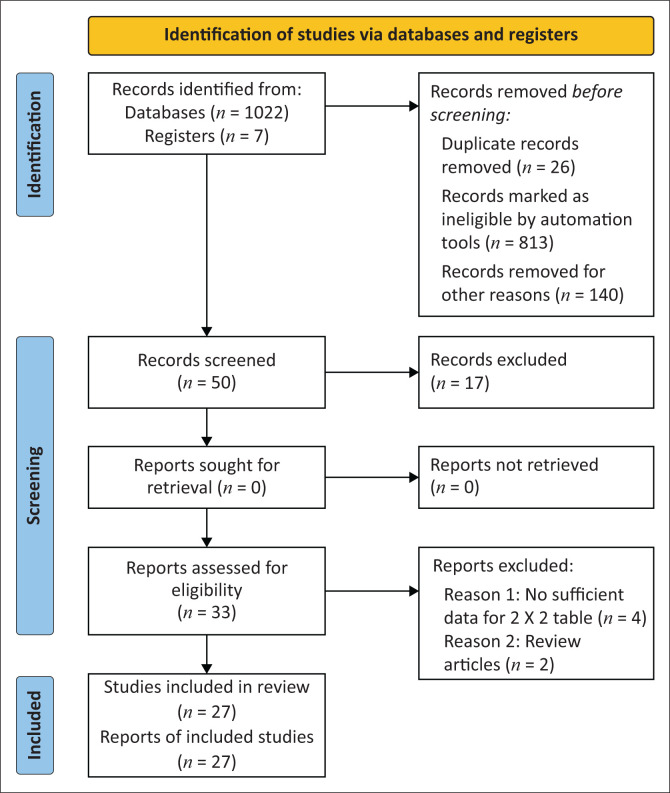
The Preferred Reporting Items for Systematic Reviews and Meta-Analyses (PRISMA) 2020 flow diagram.

Two of the investigators screened full-text articles independently through careful reading of the title and abstract, for eligibility for use in the study to minimise bias in selection. The remaining three authors independently evaluated the quality of the studies against the checklist. Any discrepancies in the inclusion of initially screened studies were resolved through discussion and, where needed, by a third reviewer. Any rejected studies were documented. The overall study selection process is presented using the Preferred Reporting Items for Systematic Reviews and Meta-Analyses statement flow chart^13^ (Figure 1).

### Data extraction and quality assessment

After the appropriate articles were selected, data were extracted independently by two of the investigators using a data extraction template and presented using Microsoft Word 2016 containing author and year, participants, country, index test, reference test, and target sequence for detection of *T. vaginalis* DNA (Table 1).

**TABLE 1:** Characteristics of included studies.

Authors publication year and reference number	Country	Total number of samples (N)	Reference test: Culture	Index test: RT-PCR	Manufacturer	Target sequence
Alikhani et al., 2021^26^	Iran	1765	-	Nested PCR	-	Actin gene
Bandea et al., 2013^27^	United States	406	Culture	*Trichomonas vaginalis* in our nested PCR assay	-	*Trichomonas* 5.8S rRNA gene
Bui et al., 2023^28^	Vietnam	535	-	Multiplex RT-PCR double quenched TaqMan probe assay	-	Fw-TV Rv-TV P-TV
Caliendo et al., 2005^29^	United States	524	Culture Diamond’s broth or Tricosal medium	BDProbeTec TaqMan1 Universal Master Mix	Applied Biosystems manufactured by Roche Branchburg, New Jersey, United States	18S ribosomal DNA gene
Chetty et al., 2020^30^	South Africa	362	-	TaqMan probe assay Pr04646256_s1	-	alpha tubulin 1 gene of *T. vaginalis*
Field et al., 2018^31^	United Kingdom	2559	Culture	TaqMan-based RT-PCR	-	*T. vaginalis* β-tubulin gene
Gaydos et al., 2017^32^	United States and Canada	990	Culture	Aptima TV	GenProbe/Hologic San Diego, California, United States	*T. vaginalis* 18S rRNA
Gaydos et al., 2006^33^	United States	321	-	GenProbe Transcription-Mediated Amplification TMA *T. vaginalis* Assay	-	*T. vaginalis* 16S rRNA gene target
Getman et al., 2011^2^	United States	3343	Culture	Aptima *T. vaginalis*	GenProbe/Hologic San Diego, California, United States	*T. vaginalis* 18S rRNA.
Goo et al., 2016^34^	South Korea	621	-	M-PCR	-	*T. vaginalis* β-tubulin gene
Hathorn et al., 2014^35^	United Kingdom	2056	Diamond’s culture medium	GenProbe Aptima TV assay	GenProbe/Hologic San Diego, California, United States	*T. vaginalis* 18S rRNA
Huh et al., 2018^36^	South Korea	1106	-	STD II MG/ MH/TV Multiplex RT-PCR Kit	-	SC STD5-HEX
Iddawela et al., 2021^37^	Sri Lanka	272	Culture	Repetitive DNA	-	TVK3/TVK7
Jordan 2001^38^	United States	552	Culture Diamond’s broth	TaqMan-based PCR	-	*T. vaginalis* β-tubulin gene
Lawing 2000^39^	United States	190	Culture	RT-PCR	-	*T. vaginalis* β-tubulin gene
Morris et al., 2023^40^	United States	1532	-	Aptima *Trichomonas vaginalis* Assays and BD ProbeTec™ *Trichomonas vaginalis* Qx Assay	BD ProbeTec™	*T. vaginalis* 18S rRNA
Nabweyambo et al., 2017^41^	Uganda	150	Culture	Gene Amp PCR System 9700 Thermocycler	Applied Biosystems Inc.	AP65 adhesin genes of *T. vaginalis*
Abraham Niehaus et al., 2021^42^	South Africa	250	Culture	TaqMan Probes	Thermo Fisher Scientific Waltham, Massachusetts, United States	*T. vaginalis* β-tubulin gene
Perazzi et al., 2016^43^	Argentina	386	Culture	TaqMan-based PCR	-	*T. vaginalis* β-tubulin gene
Pillay et al., 2007^44^	South Africa	119	Culture	TaqMan-based RT-PCR	-	bTUB β-tubulin gene
Price et al., 2018^45^	United States	359	Culture	Xpert^®^ TV	Cepheid	-
Saha 2020^46^	India	204	-	AmpliSens Russia	-	TVK3 and TVK7
Salazar et al., 2019^47^	Spain	237	Culture	The Allplex™ STI Essential assay Seegene^®^ is based on a multiplex RT-PCR method	Seegene^®^	-
Saleh et al., 2014^48^	Sudan	297	Culture Diamond’s media	TaqMan	Applied Biosystems-Roche Branchburg, New Jersey, United States	*T. vaginalis* β-tubulin gene
Schirm et al., 2007^11^	The Netherlands	1978	Culture	TaqMan primer/probe	-	Beta tubulin gene
Souza et al., 2013^49^	Brazil	556	Culture	M-PCR	-	-
Sutcliffe et al., 2010^50^	United States	1230	Culture	BTUB FRET RT-PCR	-	*T. vaginalis* β-tubulin gene

Note: Please see full reference list of this article https://doi.org/10.4102/ajlm.v14i1.2522

BTUB, β-tubulin gene; FRET, fluorescence resonance energy transfer; MG, *Mycoplasma genitalium*; MH, *Mycoplasma hominis*; M-PCR, multiplex polymerase chain reaction; PRC, polymerase chain reaction; RT-PCR real-time polymerase chain reaction; STD, sexually transmitted disease; STI, sexually transmitted infection; TV, *Trichomonas vaginalis*.

**FIGURE 2:**
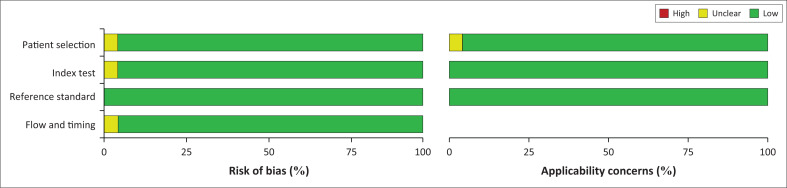
Risk of bias and applicability concerns graph: Review of authors’ judgements about each domain presented as percentages across included studies.

The methodological quality for the included studies was assessed independently according to the four domains (patient selection, index test, reference standard, and flow and timing) of the QUADAS-2 tool (Figure 2).^14^ The study QUADAS-2 quality criteria are presented in Online Supplementary Text 3.

### Data synthesis and meta-analysis

Measures of test accuracy were computed using standard methods recommended for meta-analysis of diagnostic studies. These are sensitivity, specificity, negative likelihood ratio (NLR), positive likelihood ratio (PLR), diagnostic odds ratio (DOR), and 95% confidence intervals (CI).^17,18,19^ The 2 × 2 data (true positives, false positives, true negatives, and false negatives) were extracted directly from source papers.

The DerSimonian-Laird random-effect model was used to assess the overall accuracy and DOR, which accounts for both random error (within-study variability) and heterogeneity (between-study variability). A bivariate model was used to estimate the area under the summary receiver operating characteristic (SROC) curve.^18,19^ The model uses the correlation between binary tests (sensitivity and specificity) and potential threshold effects. These measures were pooled using the random-effects model.^18,19^ Individual articles in the meta-analysis contributed a pair of numbers, sensitivity and specificity, and summarised their joint distribution using an SROC curve. A global measure of the overall performance of the test employs the area under the SROC curve, the value of 1 indicates the perfect discriminatory ability of the test, while the curve value of 0.5 means that the test does not have a discriminating ability.^18,19^ We analysed data using Meta-DiSC (version 1.4; http://www.hrc.es/investigacion/metadisc_en.htm) and Reviewing Manager version 5.4 (Cochrane Collaboration, Oxford, United Kingdom).^19,20,21^ The data were displayed graphically on forest plots and SROC plots.^22^ Since publication bias is not recommended in the meta-analysis for diagnostic test accuracy study, we did not evaluate it.^23^ No *p*-value for authors and publishers of diagnostic accuracy study was used, since they do not test the hypothesis that may influence decisions about publication based on the statistical significance of the results.^24^

### Heterogeneity

The heterogeneity tests for the included studies were explored with chi-squared (*χ*^*2*^) and I-squared (*I*^*2*^) statistics. Stratified or subgroup analyses were used to investigate the source of heterogeneity. The studies of RT-PCR assays across continents (America, Africa, Asia-Middle East, and Europe [Table 1, Table 2, Online Supplementary Table 1]) were specified a priori as potential sources of heterogeneity. The interpretations of heterogeneities among the studies are: *I*^*2*^ = 0, no heterogeneity; *I*^*2*^ < 25, low heterogeneity; *I*^*2*^ < 50, moderate heterogeneity; *I*^*2*^ < 75, high heterogeneity; and *I*^*2*^ < 90 considerable heterogeneity.^25^ They were also assessed visually, with forest plots and SROC curves with 95% prediction regions.

**TABLE 2:** Summary of statistical results for trichomoniasis clinical samples.

Test property	Summary of measure test accuracy†		Test of heterogeneity		
	Score	95% CI	*χ* ^*2*^ ‡	*I* ^*2*^	*p*
Sensitivity	99%	99–100	46.19	43.7	< 0.009
Specificity	99%	99–100	197.18	86.8	< 0.001
Positive likelihood ratio	350.67	167.42–734.49	159.14	83.70	< 0.001
Negative likelihood ratio	0.018	0.009–0.033	50.71	48.7	< 0.003
Diagnostic odds ratio	23 064.10	8532.1–62 346.8	68.07	61.8	< 0.001

Note: number of studies = 27; number of specimens = 22 472; area under receiver operating characteristics curve = 0.99.

*χ*^2^, chi-squared; *d.f.*, degree of freedom; *I*^2^, *I*-squared; CI, confidence interval.

† , random-effects model;

‡ , *d.f.* = 26.

## Results

### Study selection

A total of 1022 articles were identified through the major electronic databases and other potentially relevant sources. From all identified studies, 50 articles were selected based on their relevance to the study topic. An additional seven studies were identified from grey literature and references of full-text articles. After screening all the titles and abstracts, removing the duplicates, and excluding the ineligible studies, 27 articles (22 742 samples/patients)^26,27,28,29,30,31,32,33,34,35,36,37,38,39,40,41,42,43,44,45,46,47,48,49,50^ were selected for full-text review and meta-analysis (Figure 1).

### Characteristics of the included studies

In this systematic review and meta-analysis, 22 742 clinical samples obtained from 15 countries were included. The summary of the main characteristics of the included studies and the types of RT-PCR-based assays used is shown in Table 1. There were 13 studies from America, 5 studies from Africa, 5 studies from Asia/Middle East, and 4 studies from Europe.

The overall study quality assessment and methodological quality of studies by the QUADAS-2 tool are presented in Online Supplementary Figure 1 and Online Supplementary Figure 2. It showed a low risk of bias, except for studies using a case-control design. The methodological quality of studies (assessed by the QUADAS-2 tool) was generally high, with 27 of the studies meeting all four domains of the criteria (Figure 2). The majority of the included studies used the principle of RT-PCR assay as the index test, demonstrating culture-based assay as the reference test.

### Meta-analysis

Meta-analysis results were presented as 95% CI values for samples as follows: overall sensitivity 99% (95% CI 99–100) and specificity 99% (95% CI 99–100). Area under the curve (AUC) of the receiver operating characteristics was 0.99 for samples. The summary estimates of trichomoniasis for heterogeneity with chi-squared (*χ*^2^) using 95% CI were 46.19 (sensitivity), 197.18 (specificity), 159.14 (PLR), 50.71 (NLR), and 68.07 (DOR), with *p* < 0, indicating significant heterogeneity across studies. *I*^2^ was between 43.7% and 86.8%, showing a significant heterogeneity. There were considerable heterogeneities from the reviewed studies (Table 2, Online Supplementary Figure 3, Online Supplementary Figure 4, Online Supplementary Figure 5, and Online Supplementary Figure 6).

### Subgroup analyses of real-time polymerase chain reaction-based assay of trichomoniasis across the continents

Subgroup analyses were assessed by sources of data for these graders as seen below. An important note for all groups is that a test with perfect discrimination has a receiver operating characteristic curve that passes through the upper left corner (100% sensitivity, 100% specificity). The closer the receiver operating characteristic curve to the upper left corner, the higher the overall accuracy of the test.

With the America subgroup (Table 1) as the RT-PCR-based assay (13 studies, 10 796 specimens), the results were as follows: sensitivity 99% (95% CI 98–100), specificity 100% (95% CI, 99–100), and AUC 0.99. The summary estimates of the performance of RT-PCR-based assay in America heterogeneity, with chi-squared (*χ*^2^) using 95% CI, were 32.20 (sensitivity), 82.52 (specificity), 46.44 (PLR), 37.20 (NLR), and 43.06 (DOR), with *p* ≤ 0.001, indicating significant heterogeneity across studies. *I*^*2*^ was between 62.7% and 85.50%, showing significant heterogeneity. There was considerable heterogeneity for the subgroup analysis by RT-PCR-based assay in America (Online Supplementary Table 1; Online Supplementary Figure 7, Panels A–F).

With the Africa subgroup (Table 1) as the RT-PCR-based assay (5 studies, 1178 specimens), the results were as follows: sensitivity 99% (95% CI 99–100), specificity 99% (95% CI, 97–99), and AUC 0.99. The summary estimates of performance of RT-PCR-based assay in Africa heterogeneity, with chi-squared (*χ*^*2*^) using 95% CI, were 7.10 (sensitivity), 34.88 (specificity), 27.77 (PLR), 6.09 (NLR), and 5.42 (DOR), with *p* ≤ 0.001–0.247, indicating significant heterogeneity across studies. *I*^*2*^ was between 26.2% and 88.50%, showing significant heterogeneity. There was considerable heterogeneity for the subgroup analysis by RT-PCR-based assay in Africa (Online Supplementary Table 1; Online Supplementary Figure 8, Panels A–F).

With Asia/Middle East subgroup (Table 1) as the RT-PCR-based assay (5 studies, 3967 specimens), the results were as follows: sensitivity 100% (95% CI 98–100), specificity 100% (95% CI, 100–100), and AUC 0.99. The summary estimates of the performance of RT-PCR-based assay in Asia/Middle East heterogeneity, with chi-squared (*χ*^*2*^) using 95% CI, were 0.00 (sensitivity), 0.00 (specificity), 1.95 (PLR), 4.58 (NLR), and 3.99 (DOR), with *p* = 1, indicating significant heterogeneity across studies. *I*^*2*^ was between 0.00% and 12.70%, showing mild heterogeneity. There was considerable heterogeneity for the subgroup analysis by RT-PCR-based assay in Asia/Middle East (Online Supplementary Table 1; Online Supplementary Figure 9, Panels A–F).

With the Europe subgroup (Table 1) as the RT-PCR-based assay (4 studies, 6830 specimens), the results were as follows: sensitivity 100 (95% CI 97–100), specificity 100 (95% CI, 99–100), and AUC 0.99. The summary estimates of the performance of RT-PCR-based assay in Europe subgroup heterogeneity, with chi-squared (*χ*^*2*^) using 95% CI, were 0.00 (sensitivity), 0.00 (specificity), 2.04 (PLR), 2.91 (NLR), and 3.22 (DOR), with *p* = 1, indicating significant heterogeneity across studies. *I*^*2*^ was between 0.00% and 6.8% showing mild heterogeneity. There was considerable heterogeneity for the subgroup analysis by RT-PCR-based assay in Europe (Online Supplementary Table 1; Online Supplementary Figure 10, Panels A–F).

## Discussion

The primary aim of this study was to conduct a systematic review and meta-analysis of the relevant literature to synthesise evidence for the accuracy of RT-PCR-based assays for the diagnosis of trichomoniasis from clinical samples among women.

*Trichomonas vaginalis* infection is the most common non-viral STD worldwide; only genital human papillomavirus is more prevalent.^51^ Diagnosing the aetiology of most STDs using culture methods is infamously difficult and can take several days to complete. The most widely used diagnostic test for vaginal trichomoniasis remains the wet-mount microscopy. Culture-based assay remains the ‘gold standard’ to detect *T. vaginalis.* For the laboratory diagnosis of *T. vaginalis*, the use of PCR-based assays is more sensitive than culture and wet-mount microscopy. The uptake of the RT-PCR-based assays for routine diagnosis of *T. vaginalis* infections remains a challenge in resource-limited settings.^39^ It has been reported that ‘the sensitivity of culture compared with RT-PCR assay ranges from 34.9% to 78%, while the specificity is usually 100%’.^52^ Likewise, the wet-mount microscopy specificity is usually high; however, sensitivity of 34.2% to 58.5% was reported when compared to RT-PCR assays.^53^ It was reported that, ‘RT-PCR assay is sensitive, specific, shortened turn-around time, and is reproducible, and automation of the procedure reduces hands-on time and decreases the risk of cross-contamination’.^54^

Edwards et al. reported:

The development of sensitive nucleic acid amplification tests for *T. vaginalis* has opened the possibility of testing asymptomatic patients, who often have low organism loads, undetectable with less sensitive diagnostic methods. In the UK, the cost of offering this service in a sexual health screen is thought to outweigh the benefit of detecting these asymptomatic infections, due to the relatively low prevalence of this organism in the general population. (p. 414)^55^

In this study, we have seen the evidence that using RT-PCR-based assays for the detection of *T. vaginalis* from pathological specimens among women is rapid and accurate. The review is extensive in scope and involves different RT-PCR-based assays.

Our study findings showed high specificity of 99% (95% CI 99–100), PLR of 350.67 (167.42–734.49) and NLR of 0.018 (0.009–0.033) for trichomoniasis using RT-PCR-based assays. A PLR of 350 specifies that a *T. vaginalis* infection is 350-fold more likely to be positive for an RT-PCR-based assay in comparison to patients free from the infection. Table 2 confirms that RT-PCR-based assays were excellent for the laboratory diagnosis of trichomoniasis, as shown by AUC and DOR values. Our study showed a considerable level of heterogeneity which led us to perform subgroup analyses to investigate the likely sources of heterogeneity. Factors such as the *T. vaginalis* target gene sequence, sample size, study design, clinical settings of the primary studies, and the different RT-PCR-based assays could be responsible for the variations observed across the included studies.

### Strengths and limitations

This study’s diligent methodology, adopting the Preferred Reporting Items for Systematic Reviews and Meta-Analyses guidelines, using comprehensive search strategy and assessment in this review across different search engines for identifying published and unpublished articles is an important strength. Another strength of the review is non-restriction of studies to any language, thereby reducing bias. Potential publication bias and heterogeneity were explored using the study guidelines.^56,57^ The review had some limitations that may affect its applicability, such as the inclusion of only a few studies in our subgroup analysis, which could also impact the precision of our estimation, cost-effectiveness assessment, and the likelihood of not publishing non-significant or unfavourable results.^58^ Other limitations from this review include non-generalisation of the RT-PCR-based assay performance because of the many target genes and protocols used in the included studies.

It was reported that, ‘diagnostic studies in general seem to be beset by these problems’^59^; therefore, the outcomes of this study should be interpreted with care considering the conditions, reporting, and discrepancy in study quality. ‘The use of guidelines such as the Standards for Reporting of Diagnostic Accuracy might improve the quality of reporting of primary studies’.^60^ Further work should be considered to establish a simple, efficient and cost-effective RT-PCR assay that can be adapted for *T. vaginalis* detection from clinical samples in resource-limited countries.

### Conclusion

In conclusion, we can summarise that the present study identified RT-PCR as a highly sensitive and specific diagnostic assay compared to the reference culture-based methods for the detection of *T. vaginalis.* Furthermore, the sensitivity and specificity were 99% (95% CI 99–100) confirming the RT-PCR-based assays’ accuracy for detecting trichomoniasis in clinical samples among women.

Therefore, as a result of our findings, we recommend that healthcare practitioners and policymakers in all countries adopt the use of this type of assay on a routine basis and in STD clinics, particularly in low- and middle-income countries with a high disease burden, because of its rapid results with robust and good diagnostic accuracy when used to detect *T. vaginalis* in the clinical samples of both symptomatic and asymptomatic women.
